# Influence of CT dose reduction on AI-driven malignancy estimation of incidental pulmonary nodules

**DOI:** 10.1007/s00330-023-10348-1

**Published:** 2023-10-23

**Authors:** Alan A. Peters, Justin B. Solomon, Oyunbileg von Stackelberg, Ehsan Samei, Njood Alsaihati, Waldo Valenzuela, Manuel Debic, Christian Heidt, Adrian T. Huber, Andreas Christe, Johannes T. Heverhagen, Hans-Ulrich Kauczor, Claus P. Heussel, Lukas Ebner, Mark O. Wielpütz

**Affiliations:** 1 Diagnostic and Interventional Radiology, Heidelberg University Hospital, Im Neuenheimer Feld 420, 69120 Heidelberg, Germany; 2 Department of Diagnostic, Interventional and Pediatric Radiology, Inselspital, Bern University Hospital, University of Bern, Freiburgstrasse, 3010 Bern, Switzerland; 3 Translational Lung Research Center Heidelberg (TLRC), German Center for Lung Research (DZL), Im Neuenheimer Feld 156, 69120 Heidelberg, Germany; 4 Department of Diagnostic and Interventional Radiology With Nuclear Medicine, Thoraxklinik at University of Heidelberg, Röntgenstraße 1, 69126 Heidelberg, Germany; 5 Carl E. Ravin Advanced Imaging Laboratories, Medical Physics Graduate Program, Clinical Imaging Physics Group, Department of Radiology, Duke University Medical Center, Durham, NC USA; 6 University Institute for Diagnostic and Interventional Neuroradiology, Inselspital, Bern University Hospital, University of Bern, Freiburgstrasse, 3010 Bern, Switzerland; 7 Department of BioMedical Research, Experimental Radiology, University of Bern, Bern, Switzerland; 8 Department of Radiology, The Ohio State University, Columbus, OH USA

**Keywords:** Artificial intelligence, Lung neoplasms, Computer simulation, Radiation dosage

## Abstract

**Objectives:**

The purpose of this study was to determine the influence of dose reduction on a commercially available lung cancer prediction convolutional neuronal network (LCP-CNN).

**Methods:**

CT scans from a cohort provided by the local lung cancer center (*n* = 218) with confirmed pulmonary malignancies and their corresponding reduced dose simulations (25% and 5% dose) were subjected to the LCP-CNN. The resulting LCP scores (scale 1–10, increasing malignancy risk) and the proportion of correctly classified nodules were compared. The cohort was divided into a low-, medium-, and high-risk group based on the respective LCP scores; shifts between the groups were studied to evaluate the potential impact on nodule management. Two different malignancy risk score thresholds were analyzed: a higher threshold of ≥ 9 (“rule-in” approach) and a lower threshold of > 4 (“rule-out” approach).

**Results:**

In total, 169 patients with 196 nodules could be included (mean age ± SD, 64.5 ± 9.2 year; 49% females). Mean LCP scores for original, 25% and 5% dose levels were 8.5 ± 1.7, 8.4 ± 1.7 (*p* > 0.05 vs. original dose) and 8.2 ± 1.9 (*p* < 0.05 vs. original dose), respectively. The proportion of correctly classified nodules with the “rule-in” approach decreased with simulated dose reduction from 58.2 to 56.1% (*p* = 0.34) and to 52.0% for the respective dose levels (*p* = 0.01). For the “rule-out” approach the respective values were 95.9%, 96.4%, and 94.4% (*p* = 0.12). When reducing the original dose to 25%/5%, eight/twenty-two nodules shifted to a lower, five/seven nodules to a higher malignancy risk group.

**Conclusion:**

CT dose reduction may affect the analyzed LCP-CNN regarding the classification of pulmonary malignancies and potentially alter pulmonary nodule management.

**Clinical relevance statement:**

Utilization of a “rule-out” approach with a lower malignancy risk threshold prevents underestimation of the nodule malignancy risk for the analyzed software, especially in high-risk cohorts.

**Key Points:**

*• LCP-CNN may be affected by CT image parameters such as noise resulting from low-dose CT acquisitions.*

*• CT dose reduction can alter pulmonary nodule management recommendations by affecting the outcome of the LCP-CNN.*

*• Utilization of a lower malignancy risk threshold prevents underestimation of pulmonary malignancies in high-risk cohorts.*

**Supplementary Information:**

The online version contains supplementary material available at 10.1007/s00330-023-10348-1.

## Introduction

Pulmonary nodules are a frequent incidental finding on chest computed tomography (CT) with a substantially increasing incidence in the past three decades, mainly driven by technical improvements such as the introduction of spiral CT, increasing number of screening examinations and the widespread dissemination of CT scanners. In the literature, the proportion of chest CT scans containing pulmonary nodules varies between 15 and 50% [1, 2]. Management guidelines for incidental as well as screening-detected nodules are currently based on the nodule size, respectively the nodule volume, in combination with the individual risk profile of the patient [2–5].

Being able to accurately predict malignancy in pulmonary nodules in general would not only reduce the number of unnecessary follow-up examinations or invasive interventions, but also reduce the timespan to confirm the diagnosis for the patient. This could possibly enable curative treatment of early-stage lung cancers and hereby improve the overall survival of the patients [6].

To aid risk prediction in pulmonary nodules, various malignancy estimation models based on radiological and clinical parameters have been developed [7, 8]. One of the most renowned ones is the logistic regression model developed by the Brock University [7]. Although initially being designed for screening examinations, it has been validated successfully on cohorts with incidental pulmonary nodules in the past as well [9–11]. However, there are concerns about the use of such prediction models in clinical practice. For example, there is considerable variation between different observers regarding the data input such as precise nodule measurement or classification of the nodule borders [12, 13]. Vachani et al furthermore raised concerns regarding a potential overestimation of nodule malignancy caused by differing underlying cancer prevalence of the respective analyzed cohorts [14].

One critical step to surpass inaccuracies in radiological lesion assessment is the utilization of computer-aided diagnostic (CAD) systems. CAD systems support radiologists with detecting and classifying nodules by a reduction of inter-reader variability [15–17]. Furthermore, artificial intelligence (AI) systems in general and deep learning (DL)–based malignancy risk stratification in particular are increasingly investigated for their capability to aid correct nodule classification by malignancy prediction. These systems interact directly with the image and patient information without the need for manual data input or reader interaction. The lung cancer prediction convolutional neuronal network (LCP-CNN) evaluated in this study creates an individual malignancy risk score for each assessed nodule [18]. It was specifically designed to identify benign nodules in order to avoid unnecessary follow-up examinations [19]. Several studies have already assessed the potential of this algorithm, some of them hinting at its superiority over the established risk models [19–22]. However, in view of the increasing implementation of such systems in daily clinical practice, there is no consensus on how to use them for actual patient management, nor have they been implemented in the abovementioned guidelines. One major concern about DL-based systems is the generalizability from the available training data to all clinical scenarios encompassing local epidemiological variations, patient factors such as age, underlying lung disease such as emphysema or fibrosis, and technical bias such as CT manufacturer, CT technology (e.g., single or dual source, dual-layer detector, photon counting), scanning parameters, and CT dose. Since the presentation of a pulmonary lesion will vary with these factors, DL-based algorithms may be affected in their performance. Wichmann and colleagues have therefore recommended training the algorithms with data sets from different sites and vendors in order to avoid problems such as the disease prevalence bias or technical bias [23].

The effects of dose reduction on a DL-based CAD system have already been assessed in previous studies utilizing chest phantoms [24, 25] and have been validated in relatively small clinical cohorts as well [26, 27] but still demand verification in larger cohorts over a wide variability of CT scanners and vendors. Due to the fact that the mentioned software was designed to identify benign lesions [19], the goal was to assess the limits and false-negative rate of the software by using a cohort of proven malignancies.

The aims of this study were to assess (1) the effects of dose reduction on a deep learning-based malignancy risk stratification system using a cohort of patients with incidental lung malignancies and (2) the effects on lesion management caused by dose reduction.

## Methods/materials

### Study cohort

This study was approved by the local ethics committee and conducted in accordance with the principles of the Declaration of Helsinki. For this retrospective study, a patient selection from the local lung cancer center archive was used, consisting of 218 patients. This selection consisted of patients with histologically proven T1 lung cancers (biopsy or resection), who received the initial chest CT scan between 2013 and 2017 (Table 1).

**Table 1:** Patient and nodule characteristics

Sex (f/m)	68/101
Age [years, mean (SD)]	64.5 (9.2)
Other radiological pulmonary diagnoses, *n* (%)	
Emphysema	115 (58.7%)
Fibrosis	1 (0.5%)
Congestion	7 (3.6%)
Pleural effusion	10 (5.1%)
Pneumonia	28 (13.3%)
Atelectasis	20 (10.2%)
Bronchitis	151 (77.0%)
SAD	30 (15.3%)
Postoperative status	7 (3.6%)
Nodule localization, *n* (%)	
Right upper lobe	59 (30.1%)
Middle lobe	11 (5.6%)
Right lower lobe	43 (21.9%)
Left upper lobe	43 (21.9%)
Left lower lobe	32 (16.3%)
Central bronchi	8 (4.1%)
Nodule attenuation and size categories, *n* (%)	
Solid	157 (80.1%)
4–6 mm	7 (3.6%)
> 6–8 mm	9 (4.6%)
> 8–15 mm	56 (28.6%)
> 15–30 mm	85 (43.6%)
Part-solid	28 (14.3%)
< 6 mm	8 (4.1%)
≥ 6 mm	20 (10.2%)
Ground-glass	11 (5.6%)
< 30 mm	11 (5.6%)
> 30 mm	-
Type of malignancy, *n* (%)	
Adenocarcinoma	113 (57.7%)
SCC	47 (24.0%)
NET	18 (9.2%)
SCLC	6 (3.1%)
NSCLC	5 (2.6%)
Metastasis	3 (1.5%)
Other*	4 (2.0%)

*NET* neuroendocrine tumor, *(N)SCLC* (non-)small-cell lung cancer, *SAD* small airway disease, *SCC* squamous cell carcinoma

^*^Other entities: spindle cell carcinoma (*n* = 1), adenoid cystic carcinoma (*n* = 1), inflammatory fibroblastic tumor (*n* = 1), clear cell tumor (*n* = 1)

### Computed tomography scans and virtual CT dose reduction simulation

The 218 CT examinations of the primary cohort (80% with contrast media, *n* = 175) originated from over 20 different sites with five different CT vendors (Siemens, *n* = 130; Philips, *n* = 34; GE, *n* = 28; Toshiba, *n* = 25; Canon, *n* = 1). The acquired minimum slice thickness varied from 0.5 to 4 mm, the majority of scans having a minimum slice thickness of ≤ 1.5 mm (66.8%, Table 2). The reconstruction algorithms included filtered-back projection (*n* = 98) as well as iterative reconstruction (*n* = 71). Scan volumes varied from chest-only acquisitions to whole-body examinations. The mean effective doses were 4.4 mSv, 12.8 mSv, 9.0 mSv, and 4.2 mSv for the chest-only acquisitions (*n* = 101), chest plus neck or abdomen acquisitions (*n* = 35), whole-body acquisitions (*n* = 3), and PET/CT scans (*n* = 30), respectively. The CT examinations were transferred to a dedicated post-processing imaging lab specialized on LDCT simulations (Ravin Advanced Imaging (RAI) Lab, Duke University). The reduced dose simulations were produced by adding statistical noise to the images using a previously described CT image-based noise addition tool [28]. Two low-dose simulation levels were created for each CT scan, which led to three different dose levels for each examination: Original, 25% and 5% dose level. The 25% and 5% dose levels were chosen to resemble low-dose (1–2 mSv) and ultralow-dose (0.1–0.2 mSv) chest CT scans [29].

**Table 2:** CT scan parameters

Dose parameters of the original scans, mean (SD)	
DLP, mGycm	
Chest only (*n* = 101)	315.2 (238.3)
Chest plus neck/abdomen (*n* = 35)	915.3 (633.5)
Whole-body acquisition (*n* = 3)	643.7 (559.7)
PET-CT (*n* = 30)	297.5 (151.1)
CTDIvol, mGy	
Chest only (*n* = 101)	13.4 (13.3)
Chest plus neck/abdomen (*n* = 35)	26.4 (26.5)
Whole-body acquisition (*n* = 3)	20.7 (28.8)
PET-CT (*n* = 30)	4.0 (2.1)
Effective dose, mSv^#^	
Chest only (*n* = 101)	4.4 (3.3)
Chest plus neck/abdomen (*n* = 35)	12.8 (8.8)
Whole-body acquisition (*n* = 3)	9.0 (7.8)
PET-CT (*n* = 30)	4.1 (2.1)
Minimum slice thickness, *n* (%)	
0.5 mm	4 (2.4%)
0.625 mm	3 (1.8%)
0.75 mm	1 (0.6%)
0.9 mm	5 (3.0%)
1 mm	67 (39.6%)
1.25 mm	26 (15.4%)
1.5 mm	7 (4.1%)
2 mm	46 (27.2%)
2.5 mm	1 (0.6%)
3 mm	7 (4.1%)
4 mm	2 (1.2%)

*CTDIvol* volume computed tomography dose index, *DLP* dose-length-product, *PET-CT* positron emission tomography-computed tomography, *SD* standard deviation

^#^Calculated using the conversion factor of 0.014 [38]

### Lung cancer prediction convolutional neural network

The algorithm used in this study is a commercially available FDA-approved LCP-CNN, which is based on a Dense Convolutional Network, a type of deep learning CNN architecture designed for computer vision tasks [20, 21, 30]. It was trained on the National Lung Screening Trial (NLST) data. In the standard procedure of the utilized version, the respective nodule is manually marked by a radiologist, the algorithm then automatically segments the nodule and a certain perinodular region of interest, with no possibility for secondary manual adjustments. It then provides a score from 1 to 10, which can be read as a likelihood for malignancy of that specific nodule, hereby a higher score indicates a greater chance of malignancy. Kim et al proposed the division into three risk groups based on the malignancy risk thresholds according to the American College of Chest Physician guidelines: LCP score ≤ 4 (malignancy risk < 5%), LCP score 5–8 (malignancy risk 5–65%), and LCP score ≥ 9 (malignancy risk > 65%) [22, 31]. A detailed description of the LCP-CNN can be found in the supplements.

### Assessment of impact on patient management

In order to assess a possible impact of simulated dose reduction on patient management, the proportion of correctly classified nodules was compared between the three dose levels based on the two malignancy risk thresholds according to the American College of Chest Physician guidelines [5]. The guidelines propose a 5% (“rule-out”) and a 65% (“rule-in”) malignancy risk threshold, dividing the current cohort into three risk groups:

- Low risk: LCP score ≤ 4 equal to a malignancy risk < 5%
- Intermediate risk: LCP score 5–8 equal to a malignancy risk from 5 to 65%
- High risk: LCP score ≥ 9 equal to a malignancy risk > 65%

Following these categories, the definition of a “correctly” classified malignant nodule would be a score of 9 or 10 using the “rule-in”-approach and a score of > 4 using the “rule-out”-approach. For the purpose of this study, changes in these categories based on simulated dose reduction were assumed clinically relevant for patient management.

### Statistical analysis

All analyses were performed using SPSS (SPSS Statistics, IBM Corp., version 25.0.) and GraphPad Prism (GraphPad Software, Inc., version 8). Metric variables are reported as mean (standard deviation), categorical variables as absolute number (relative proportion). The LCP scores of the three dose levels were compared by using the Friedman ANOVA for paired samples, the number of correctly classified nodules by using the Cochran’s *Q* test. Cohen’s Kappa (κ) was used to assess the correlation between the LCP scores of the different dose levels. Hereby, κ was interpreted as follows: slight agreement (0 < κ ≤ 0.2), fair agreement (0.2 < κ ≤ 0.4), moderate agreement (0.4 < κ ≤ 0.6), substantial agreement (0.6 < κ ≤ 0.8), almost perfect agreement (0.8 < κ ≤ 1.0) [32]. A *p* value < 0.05 was considered statistically significant.

## Results

### Patient and nodule characteristics

**Fig. 1:**
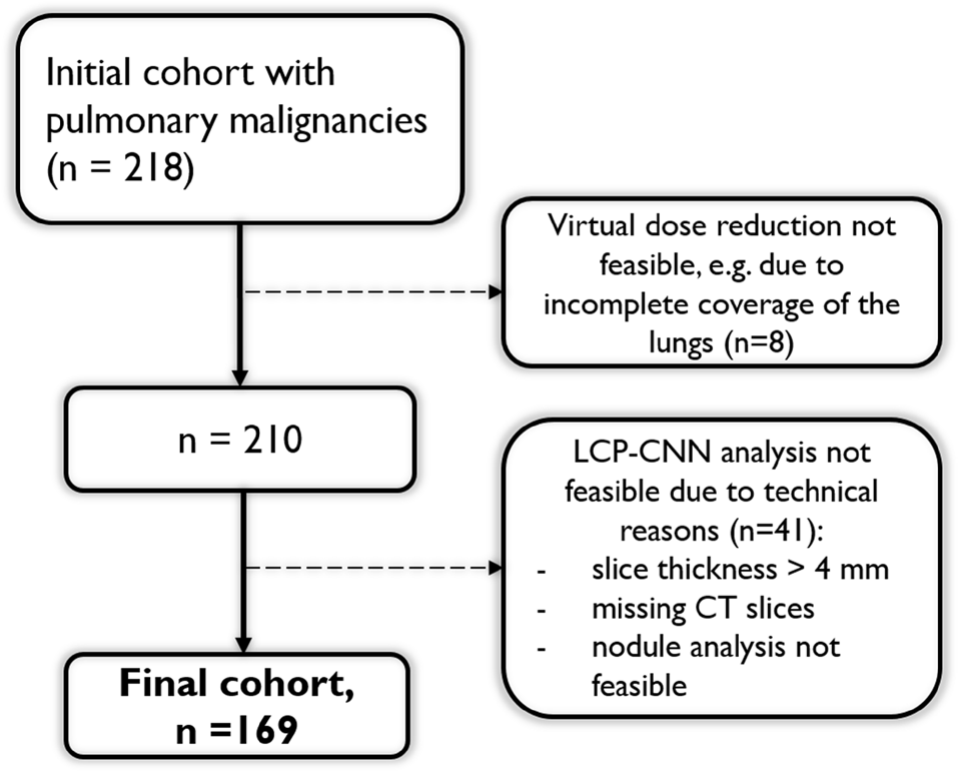
Patient flowchart

After evaluation of the primary cohort, eight patients had to be excluded because the virtual dose reduction simulation was not feasible due to technical issues such as incomplete coverage of the lungs. Another 41 patients had to be excluded because the LCP-CNN rejected to analyze the CT datasets due to restrictions, such as a slice thickness > 4 mm, missing CT slices or because the nodule segmentation was not feasible (Fig. 1).

**Fig. 2:**
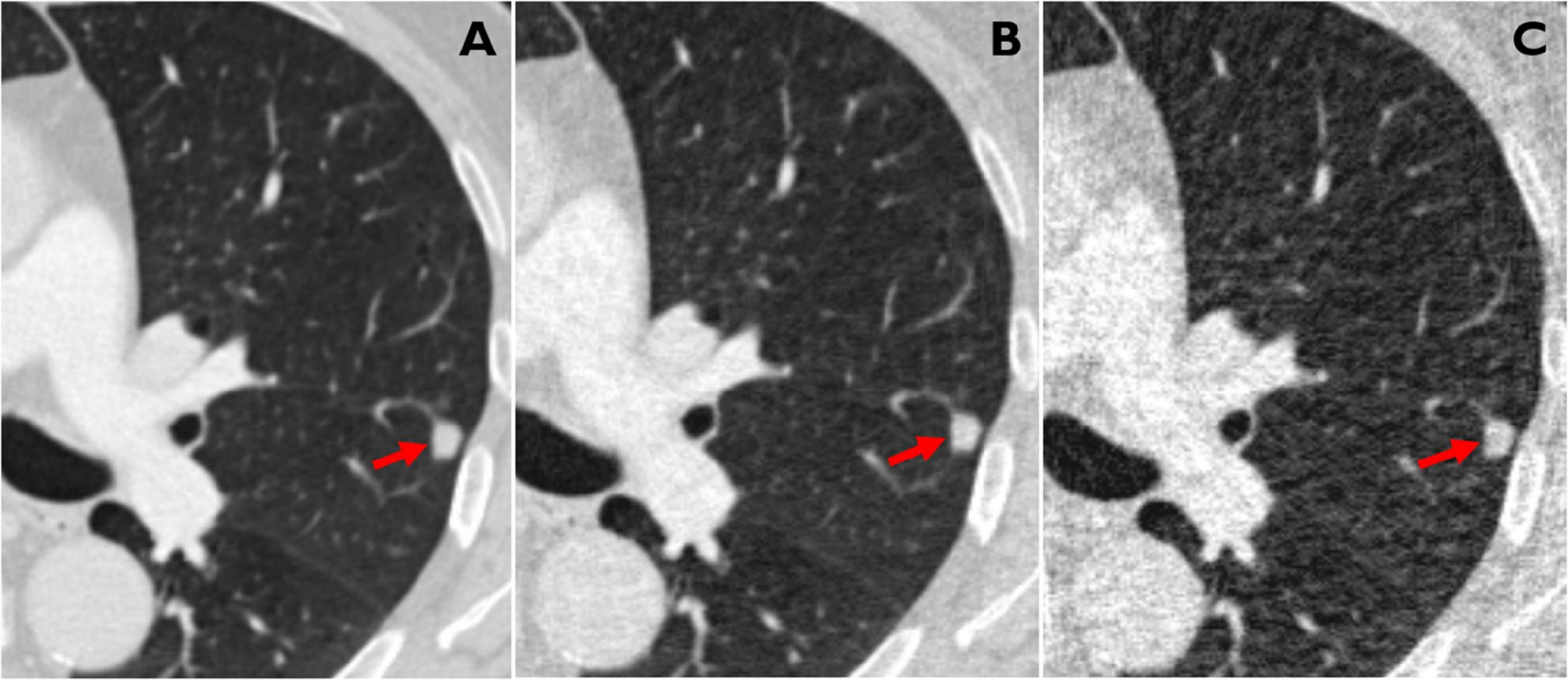
CT images of a 65-year old male patient (former smoker, 40 pack-years) depicting a small cell lung cancer (red arrow) with a diameter of 10 mm in the left upper lobe (circle) at 100% (**A**), and simulated 25% (**B**) and 5% (**C**) dose. The respective LCP scores for the three dose levels were 4, 3, and 3 (corresponding to an estimated malignancy risk < 5% for all three dose levels)

**Fig. 3:**
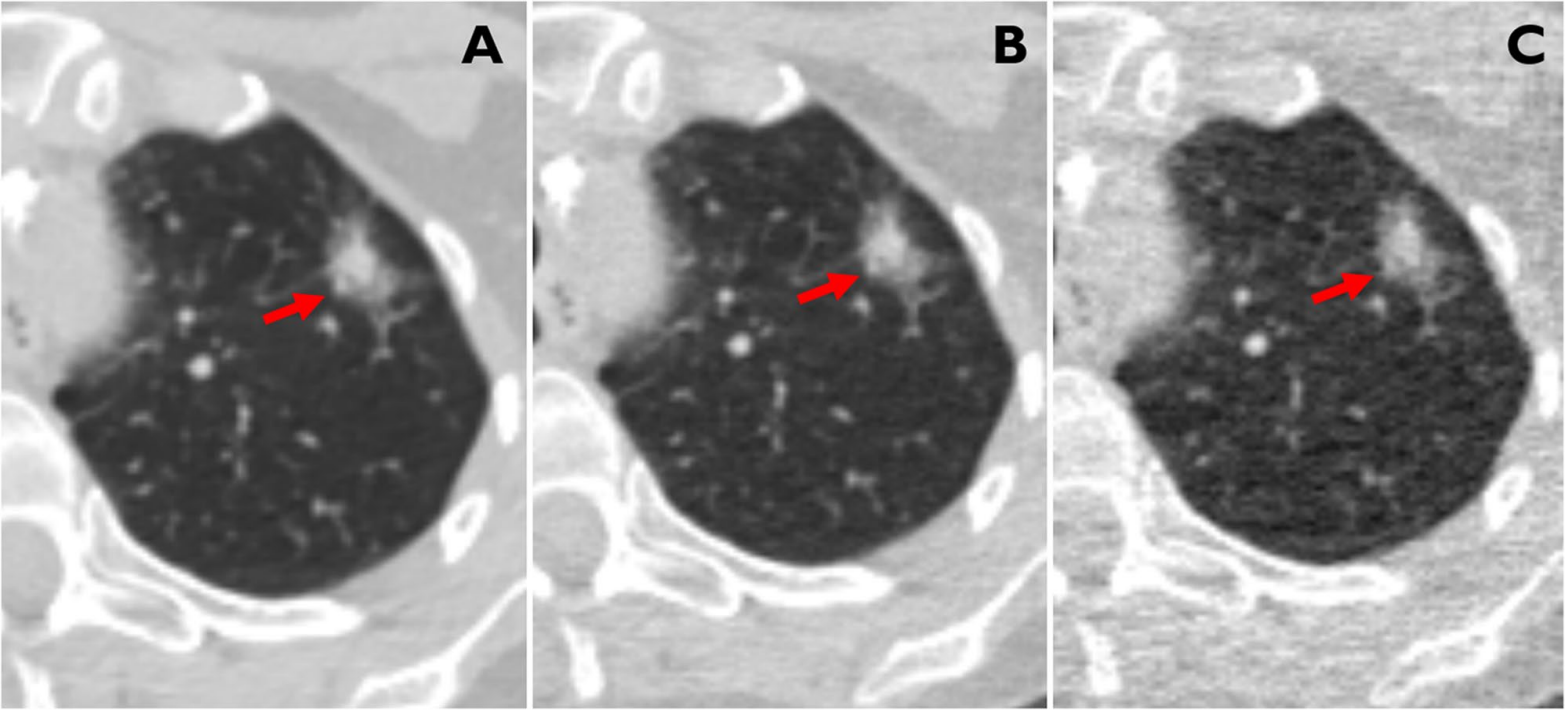
CT images of a 61-year-old female patient (longtime smoker) with an 18-mm part-solid lesion (solid part 8 mm) in the left upper lobe (red arrow) depicted with 100% (**A**), and simulated 25% (**B**) and 5% (**C**) dose. The respective LCP score for the three dose levels was 10 (corresponding to an estimated malignancy risk > 65% for all three dose levels). Biopsy showed that the tumor was an adenocarcinoma

In total, 169 patients with 196 malignancies could be included into the study (mean age ± SD, 64.5 ± 9.2 years, 49% females; Table 1, Figs. 2 and 3). One hundred eight-nine nodules were primary malignancies of the lung, three were metastases and four were other entities (spindle cell carcinoma, *n* = 1; adenoid cystic carcinoma, *n* = 1; inflammatory fibroblastic tumor, *n* = 1; clear cell tumor, *n* = 1; Table 1).

### Simulated dose reduction leads to inferior performance of the LCP-CNN

**Fig. 4:**
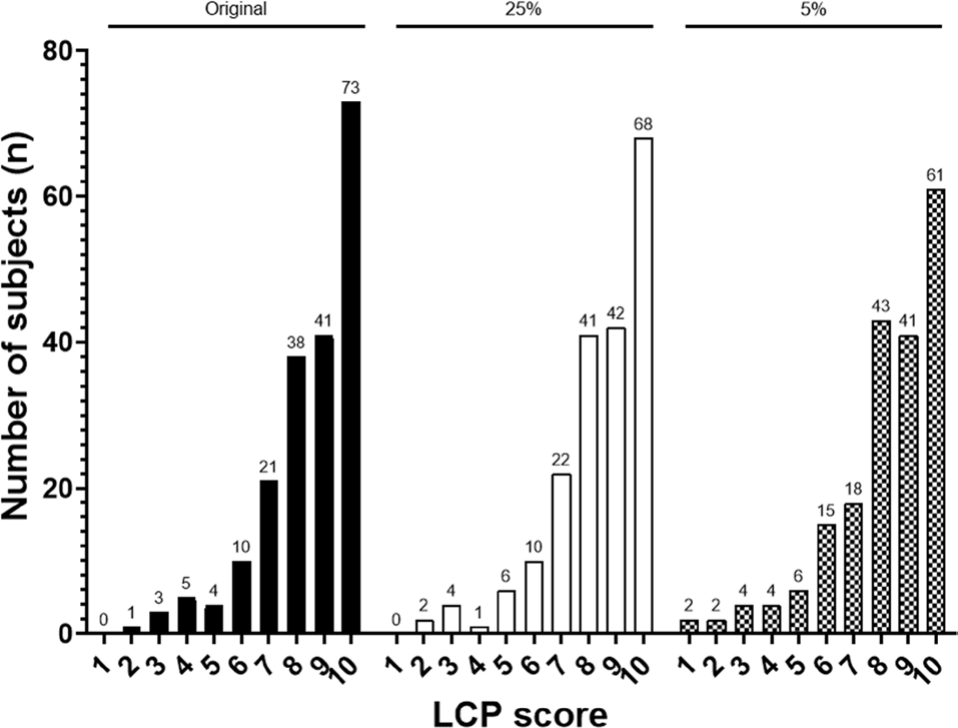
Histogram of the of Lung Cancer Prediction (LCP) scores grouped by dose level

The mean LCP scores were similar for the original dose examinations (8.5 ± 1.7) compared to the 25% dose (8.4 ± 1.7, *p* = 0.42) and higher compared to the 5% dose simulations (8.2 ± 1.9, *p* = 0.006) (Table 3, Fig. 4). The difference between 25 and 5% dose simulations did not reach statistical significance (*p* = 0.07). The correlation between the scores of the original dose and 25% dose was almost perfect (κ = 0.81) whereas the correlation between original and 5% dose was only moderate (κ = 0.54).

**Table 3:** Crosstab of Lung Cancer Prediction (LCP) scores between different dose levels

		25%-dose level LCP scores									5%-dose level LCP scores									
Original dose LCP scores		2	3	4	5	6	7	8	9	10	1	2	3	4	5	6	7	8	9	10
	2	**1**									1	**0**								
	3	1	**2**									2	**1**							
	4		1	**1**	2								2	**1**	1					
	5				**4**								1	1	**2**					
	6		1			**8**	1				1			1	1	**6**		1		
	7					2	**18**	2							1	8	**9**	4		
	8						3	**32**	2	1						1	8	**23**	6	
	9							7	**34**								2	12	**25**	2
	10								6	**67**					1			3	10	**59**

Bold indicating concordant scores

Comparison of the three dose levels using the 5%-malignancy risk threshold (“rule-out” threshold) revealed no significant differences regarding the proportion of correctly classified nodules (95.9% vs. 96.4% vs. 94.4%; *p* = 0.12). When performing the same analysis using the 65% malignancy risk threshold (”rule-in” threshold), the algorithm classified significantly less nodules as high risk nodules using the 5% dose simulations compared to the original dose (52.0% vs. 58.2%; *p* = 0.01). Of note, original dose and 25% dose level simulations yielded similar sensitivity (56.1% vs. 58.2%, *p* = 0.34).

A subgroup analysis revealed no significant correlation between the proportions of correctly classified nodules with the most frequent underlying lung pathologies (bronchitis, emphysema and small airway disease).

### Simulated dose reduction can cause reclassification of the malignancy risk group

**Fig. 5:**
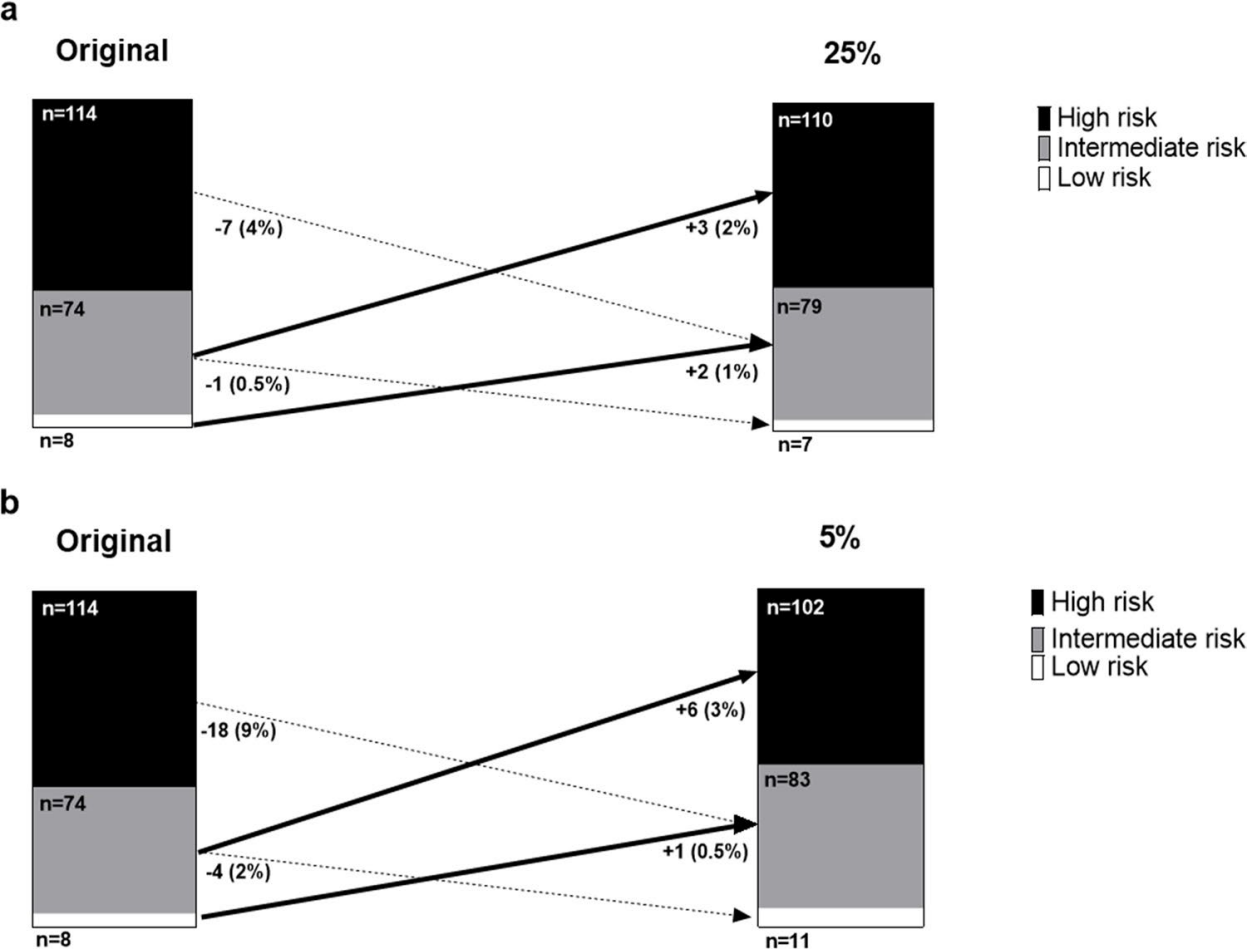
Risk group reclassification by simulated dose reduction. **a** Original vs. 25% dose. **b** Original vs. 5% dose

The distribution between the different malignancy risk groups was similar for the three dose levels (Table 4). In total, 7% (*n* = 13) of all nodules shifted to another malignancy risk group when comparing the original dose to the 25% dose simulation scans; hereby, 3% (*n* = 5) of the nodules shifted to a higher malignancy risk group, and 5% (*n* = 8) shifted to a lower risk group (Fig. 5). Regarding attenuation and size groups, 23% (*n* = 3) of the shifted nodules were subsolid, 20% (*n* = 2) of the solid nodules were < 10 mm.

**Table 4:** Risk group distribution by dose level

	Original dose	25% dose	5% dose
Low risk (< 5%), LCP score 1–4	8 (4.1%)	7 (3.6%)	11 (5.6%)
Intermediate risk (5–65%), LCP score 5–8	74 (37.8%)	79 (40.3%)	83 (42.3%)
High risk (> 65%), LCP score 9–10	114 (58.2%)	110 (56.1%)	102 (52.0%)*

^*^p = 0.012 vs. original dose

When comparing the scores of the 5% dose simulations to the scores of the original scans, 14.8% (*n* = 29) of the nodules were re-classified to another malignancy risk group; 4% (*n* = 7) shifted to a higher malignancy risk group, and 11% (*n* = 22) shifted to a lower risk group (Fig. 5).

Regarding the attenuation and size groups, 24% (*n* = 7) of the shifted nodules were subsolid, 23% (*n* = 5) of the solid nodules were < 10 mm.

## Discussion

In this study, we could demonstrate that simulated dose reduction has an effect on the performance of a DL-based malignancy risk stratification system in cohort of proven pulmonary malignancies, which may have implications regarding patient management.

The dose dependency of AI-based CAD systems has been described previously [24, 33–35]; however, the existing studies primarily focused on nodule detection rather than malignancy risk estimation. So far, the evaluation of dose reduction effects was mainly examined in lung cancer screening programs than in the context of incidental pulmonary nodules. However, recent developments such as photon counting detectors will inevitably increase the number of low-dose chest CT applied in daily clinical routine [36] and underline the relevance for incidental pulmonary malignancies. Jungblut et al for example have taken first steps in this direction by evaluating the performance of energy-integrating detector (EID)-CT trained algorithms in photon counting CT scans with reduced dose levels [37].

In our study, the LCP-CNN scored 58% (*n* = 114/196) of all malignancies as ≥ 9, which is the so-called rule-in threshold [5], in the original scans. This proportion decreased significantly to 52% when using the 5% dose simulations. These numbers are somewhat comparable to the results of Massion et al, who reported proportions of 36% and 70% correctly classified lung cancers in two different cohorts [20].

Using the so-called rule-out approach with a threshold LCP score of < 4, there were 4% (*n* = 8), 4% (*n* = 7), and 6% (*n* = 11) false-negatives observed for the original, the 25%, and the 5% dose simulation, respectively, the difference not reaching statistical significance. The corresponding values reported by Massion and colleagues for their two cohorts were 2% and 3%, respectively. These findings underline the aforementioned fact that the algorithm was primarily designed to rule out benign nodules rather than to correctly identify malignant ones. Interestingly, the differences to Massion et al were not as striking as expected, despite the fact that the current work included both types of nodule densities, solid and subsolid, as well as contrast-enhanced and non-contrast CT scans. Massion and colleagues only included non-contrast scans with solid nodules, which the software was initially trained on. Apparently, the algorithm is able to process contrast-enhanced scans and subsolid nodules with a comparable performance as well.

Regarding the effects of simulated dose reduction, it could be shown that a reduction to 25% and 5% of the original dose leads to lower mean LCP scores, at least in this specific setting, which focused solely on pulmonary malignancies. In order to elaborate the clinical relevance of these findings, the malignancy risk group shifts between the three dose levels were analyzed. Considering all upward and downward shifts, 4% (low-dose) and 9% (ultralow-dose) of all nodules were falsely categorized into the medium- instead of the high-risk group, which could hypothetically delay the correct diagnosis and timely treatment of patients. However, only 0.5% and 2% of the nodules were falsely categorized into the low-risk group after simulated dose reduction, again underlining that the LCP-CNN should rather be used in a “rule-out” approach with a low malignancy risk threshold.

This study has several limitations. First, the cohort did not fulfill the criteria the software was initially designed and approved for, since it contained contrast-enhanced scans and subsolid nodules. Despite this fact, the algorithm showed a performance in the current study comparable to the validation studies, which were in keeping with the strict admission criteria [20]. Furthermore, it seemed pertinent to evaluate the software performance not only for one specific setting, especially in front of the upcoming or already established lung cancer screening programs all over the globe, in which a wide variety of different vendors, scanner types, and scan protocols can be expected.

Second, only histologically proven malignant nodules were included in this study. This approach allowed the assessment of the systems’ false-negative rate but does not allow any statement on false-positive rates and limits the comparability with similar studies. However, due to the fact that the software was designed to rule out malignancy in pulmonary nodules instead of detecting malignancies correctly, as reported in the past already [19], it seemed more tempting to assess the software’s limits by using a cohort of proven malignancies. However, validation of our results in a cohort including a benign control group is warranted.

Third, the authors are aware that virtual dose reduction created during post processing is not a perfect substitute for low-dose or ultralow-dose CT scans. However, this approach has been validated before [28] and enabled intra-patient comparison without the need for unnecessary radiation exposure.

In conclusion, this study showed that simulated dose reduction by 75% appears to be feasible without significantly altering the outcome of the LCP-CNN. Simulated dose reduction by 95% to an ultralow-dose level potentially alters the outcome of a DL-based malignancy risk estimation system, at least in the current setting using a high-risk cohort of proven malignancies, and that this alteration may affect patient management. However, underestimation of lung cancer can be avoided by using a “rule-out” approach with a lower malignancy risk score threshold.

The next step towards clinical implementation of the software are the validation of the achieved results by repeating the analysis on a larger cohort, ideally in a prospective, randomized-controlled setting and containing both malignant and benign nodules, as well as a larger number of non-solid nodules.

### Supplementary Information

Below is the link to the electronic supplementary material.

Supplementary file1 (PDF 522 KB)

## Abbreviations

(m)Sv: (Milli)Sievert
AI: Artificial intelligence
CAD: Computer-aided diagnosis
CNN: Convolutional neural network
CT: Computed tomography
DL: Deep learning
EID: Energy-integrating detector
FDA: Food and Drug Administration
GGN: Ground-glass nodule
LCP: Lung cancer prediction
Lung-RADS: Lung Imaging Reporting and Data System
NLST: National Lung Screening Trial
RAI: Ravin Advanced Imaging

## Declarations

### Guarantor

The scientific guarantor of this publication is Dr. Peters.

### Statistics and biometry

Several authors have significant statistical expertise (AP, AH, AC, MW).

### Study subjects or cohorts overlap

The study subjects have not been reported in a previous publication.

### Methodology

• retrospective

• observational

• performed at one institution
