# Supplementary material for: Influence of CT dose reduction on AI-driven malignancy estimation of incidental pulmonary nodules
Source: Eur Radiol. 2023 Oct 23;34(5):3444–52. doi: 10.1007/s00330-023-10348-1 (PMC11126495; doi:10.1007/s00330-023-10348-1)
Supplement: Supplementary file 1 — Supplementary file1 (PDF 522 KB) [file 330_2023_10348_MOESM1_ESM.pdf]

Supplemental Material

("Influence of CT dose reduction on AI-driven malignancy estimation of incidental pulmonary nodules")

Description of the LCP-CNN

Model implementation details: The LCP-CNN is based on a DenseNet architecture with 5 dense blocks, each containing 4 composite functions BN-ReLU-Conv[1×1]-BN-ReLU-Conv[3×3]. The input to the LCP-CNN is resampled at a resolution of 0.25mm x 0.25mm x 1mm, which is augmented at training time using random cropping, flipping and rotations. A 2.5D model is used, since it was found in early development that the 3D model did not provide sufficient gains to warrant its use. This is most likely due to the heterogeneity of our dataset in terms of imaging protocols and other factors. The LCP-CNN system was pre-trained using >130,000 images selected and curated to optimally prime the network for subsequent training. Class balancing was used in the CNN training to account for the lower proportion of malignant nodules in the training dataset; without this the resulting CNN would be tuned to benign nodules. During training, the input patches are sampled from the training data so that approximately the same sample number of each class (benign and malignant) is used. The network training procedure attempts to optimize a cross-entropy loss function, and the network parameters are updated according to the ADAM optimizer until convergence. The LCP-CNN has been developed using the PyTorch framework for machine learning[1].

The LCP-CNN network was trained using solid and semi-solid nodules of at least 5mm in diameter. The training data consisted of 8% malignant nodules and 92% benign nodules, and included both screening (95%) and incidentally detected (5%) nodules. The median age of the subjects was 62 (20-90) and included a mix of US (95%) and EU data (5%). The data included females (38%) and males (62%). Only nodules that were confidently matched to a definitive diagnosis, as provided with the data, were used for training.

LCP-CNN's output is a continuous value between 0 and 1; this is mapped to an integer score between 1 and 10. This mapping was constructed by computing the raw LCP score on a dataset consisting of malignant (10%) and benign nodules (90%) but which were not used during the model training. The mapped integer score is shown to the user alongside a plot detailing the cancer prevalence in each of 10 bins for a population with a 30% cancer prevalence.

1. *Paszke A, et al. An Imperative Style, High-Performance Deep Learning Library. NIPS 2019: 8024-8035.*

Development and validation of a generic image-based noise addition method for simulating reduced dose computed tomography images with realistic noise properties

A) Tool development

The noise addition tool aims to add noise with texture and spatial non-stationary that are similar to those in real low-dose CT images. The addition procedure is performed in two domains: (a) image domain, where key features of the input CT series are extracted, and (b) projection domain, where noise is added into synthetic projections. Fig. 1 shows an overview of the simulation procedure.

The tool was built using Python programming language (Python Software Foundation, https://www.python.org/), and designed to run using either GPU or CPU systems. The tool was based on a noise addition methodologies that entails eight specific steps.

(1) The method first estimates the noise power spectrum (NPS) across CT series.[2] For each series, it also measures the global noise index (GNI) of each slice in the image domain using established techniques.[3]

(2) To account for cross-slice noise correlations, the next step smooths the estimated GNI across slices. This is achieved by applying an averaging filter that takes the average of all GNI measurements within the kernel area. The filter helps to reduce noise fluctuations, resulting in smoother GNI values across different slices.

(3) The next step involves generating a synthetic sinogram for each image slice through two sub-steps:

   a. First, it converts the Hounsfield unit (HU) to linear attenuation coefficients (µ) in the input CT image using nominal linear attenuation coefficients of water (µwater) and air (µair).

b. Second, it forward projects the linear attenuation coefficients using the ASTRA toolbox.[4,5]

(4) Following that, the sinogram noise is constructed by generating zero-mean white Gaussian noise in the projection domain. The generated noise has a spatially variable magnitude, with higher noise corresponding to projections with higher attenuation and vice versa.

(5) Once the sinogram noise is generated, the next step involves backprojecting this noise across an image matrix that matches the original CT slice. The backprojection process creates a noise-only image, which captures the spatial distribution and characteristics of the noise in the original CT series.

(6) The noise image is then filtered using NPS fit. The filtering step is performed to obtain realistic noise correlations and non-stationary noise magnitude across the field of view.

(7) Next, the noise image is scaled based on the desired dose reduction level and the estimated noise in the input CT image. This scaling process is done using the inverse power law relationship between dose (D) and noise (σ).

$$\sigma \propto D^{-\beta} \quad (1)$$

Theoretically, β is assumed to have a value of 0.5 for linear reconstruction algorithm.

(8) Finally, the scaled noise image is added to the input CT image, generating a simulated reduced-dose image.

This comprehensive noise addition process enables the generation of realistic reduced-dose CT images. The tool is designed to have minimal input requirements, allowing the user to specify certain parameters, as shown in Table 1.

Table 1. Adjustable parameters of the noise addition tool.

Parameters
Radiation dose reduction level β
μwater [1/mm]
μair [1/mm]
Number of projection angles
Number of detector elements
Radius of bowtie filter [mm]

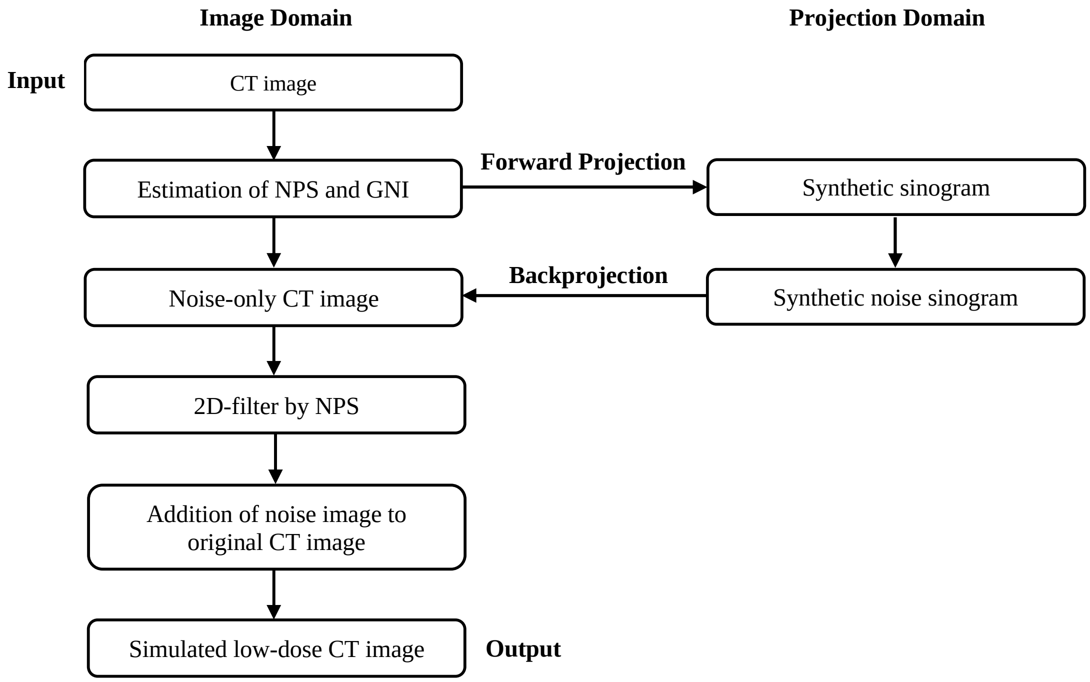

Fig. 1. Schematic overview of the noise addition software tool.

B) Validation of the noise addition tool using phantom images

Validation of the noise addition tool was performed using two phantoms. First, a uniform multi-sized image quality phantom (Mercury phantom version 3.0, Duke University)[6] to evaluate the tool performance at different diameters. Second, a thorax anthropomorphic phantom (Lungman Phantom, Kyoto Kagaku) to assess the tool in the presence of more complex structures. Both phantoms were imaged on Siemens SOMATOM Flash scanner (Siemens Healthcare) at dose levels of 40, 20, 10, and 3 mGy. Table 2 shows the acquisition parameters. The images were reconstructed with filtered back projection (FBP, Br40s and Br62s kernels), and Sinogram

affirmed iterative reconstruction (SAFIRE, Br40s kernel, strength level 3). Each scan was repeated to provide duplicate images, which were used for subtraction purposes.

Table 2. Acquisition and reconstruction settings for Mercury phantom and anthropomorphic phantom.

Scan mode	Helical	
Detector configuration	128 x 0.6 mm	
Beam width	38.4 mm	
Pitch	1	
CTDIvol	40, 20, 10, 3* mGy	
Slice thickness	3 mm	
Reconstruction algorithm	Filtered back projection	SAFIRE (IR)
Reconstruction kernel	Br40s, Br62s	Br40s\3

*The anthropomorphic phantom was only reconstructed with Br40s for 3 mGy scans

The noise addition tool was used to simulate reduced-dose images at dose levels of 20, 10, and 3 mGy based on the acquired images. From the acquired images, β values were determined empirically for each reconstruction kernel of both phantoms. These β values of 0.476, 0.418, and 0.447 were used for Br40s, Br62s, and Br40s\3, respectively, in the simulations.

To assess the accuracy of the simulated noise magnitude, a comprehensive analysis was conducted. The analysis compared the noise magnitude in the simulated reduced-dose images with the corresponding actual reduced-dose images of both phantoms. Fig. 2 provides an overview of the noise magnitude analysis. The following steps were involved in the analysis:

(1) The first step in the analysis involved subtracting the duplicate images (I_A, I_B) from each other to obtain noise-only images. To account for the error propagation from the subtraction, the noise measurements are divided by the square root of two, as follows:

$$S_R = \frac{(I_{A,R} - I_{B,R})}{\sqrt{2}} \quad (2)$$

$$S_S = \frac{(I_{A,S} - I_{B,S})}{\sqrt{2}} \quad (3)$$

Eq. (2) represents the real noise-only images (S_R), where $I_{A,R}$ and $I_{B,R}$ are the real duplicate images. Eq. (3) represents the simulated noise-only images (S_R), where $I_{A,S}$ and $I_{B,S}$ are the simulated duplicate images.

(2) From the noise images, spatial maps of the noise magnitude (σ_{map}) were generated. This procees began by applying a standard deviation filter, represented by a discrete operation h, to the noise images. Then, The resulting filter images were then multiplied by a binary mask (M) to remove the background. The noise magnitude maps were computed as $\sigma_{map,R}$ and $\sigma_{map,S}$ for the real and simulated images, respectively, as follows:

$$\sigma_{map,R} = (S_R * h) \times M \quad (4)$$

$$\sigma_{map,S} = (S_S * h) \times M \quad (5)$$

(3) Following that, the difference noise magnitude maps ($\sigma_{map,Diff}$) were obtained by subtracting the simulated noise magnitude maps ($\sigma_{map,S}$) from the real noise magnitude maps ($\sigma_{map,R}$) as shown in Eq. (6).

$$\sigma_{map,Diff} = \sigma_{map,R} - \sigma_{map,S} \quad (6)$$

(4) To assess the accuracy of the simulated noise magnitude at the individual pixel level, the relative error of the noise magnitude (δ_{pixel}) was calculated for each pixel using Eq. (7).

$$\delta_{pixel}\,(\%) = \frac{\sigma_{map,Diff}}{\sigma_{map,R}} \times 100 \quad (7)$$

(5) To evaluate the overall accuracy of the noise addition tool in simulating the noise magnitude, the relative error of the mean noise magnitude (δ_{mean}) was computed using the following:

$$\delta_{mean}\,(\%) = \frac{\bar{\sigma}_{map,R} - \bar{\sigma}_{map,S}}{\bar{\sigma}_{map,R}} \times 100 \quad (8)$$

In this equation, $\bar{\sigma}_{map,R}$ and $\bar{\sigma}_{map,S}$ represents the mean of the real and simulated masked noise magnitude maps, respectively.

The NPS was also assessed and compared between the simulated and acquired images at the corresponding dose level and reconstruction setting using an established technique.16 The NPS describes the spatial frequency distribution of the noise and is defined as the Fourier transform of the noise autocorrelation function.[7,8]

To assess the performance of the tool under low-signal conditions, several steps were taken. First the average signal level at the detector for a given image slice was calculated using Eq. (9).

$$DDI = CTDI_{vol} \times e^{-(\mu_{water} \times WED)} \quad (9)$$

This equation defines the detector-dose index (DDI), and takes into account the linear attenuation coefficient of water (μ_{water}), the water-equivalent diameter (WED) of the image slice, and the volume CT dose index ($CTDI_{vol}$). After measuring the DDI for each simulated and real image slice in both phantoms, the average noise magnitude was calculated using the previously described methodology. Then, the average noise magnitude difference between the simulated and real image slices of both phantoms was computed. Lastly, the average noise magnitude difference values were analyzed in terms of DDI, to identify the imaging conditions which the tool may produce irrelevant results.

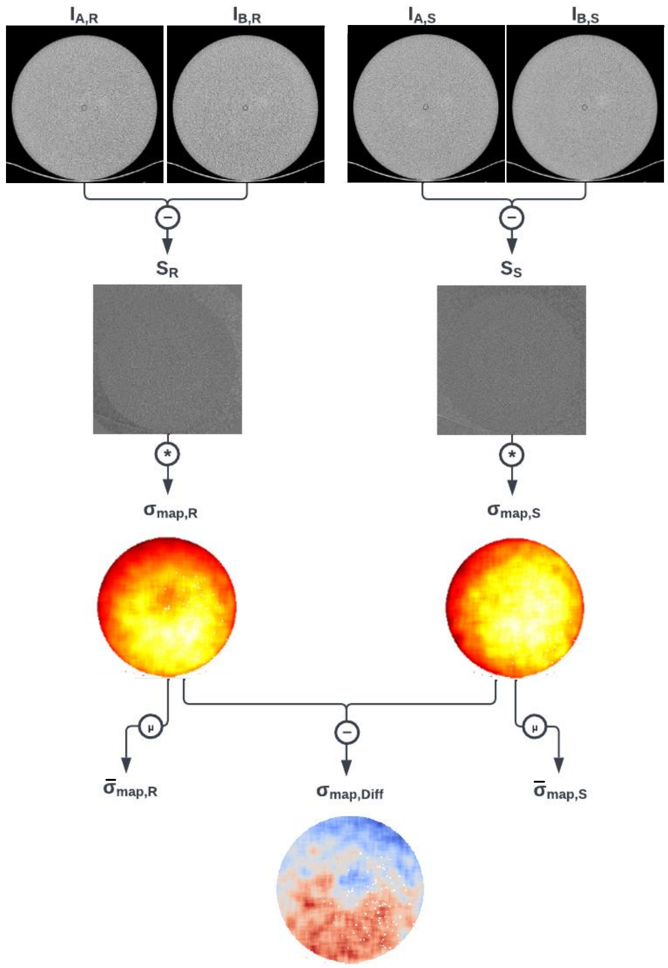

Fig. 2. Summary of the noise magnitude analysis for the phantom images.

C) Validation of the noise addition tool using patient images

Patient images contain detailed anatomical structures and textures that have an impact on physical image properties like resolution and quantum noise.[9] In contrast, phantom images lack the complex relationship between anatomical variability and image quality. Therefore, to ensure more clinically relevant validation, patient images were used to assess the performance of the noise addition tool. Patient image data were taken from a previously conducted institutional research board approved clinical trial at Duke University. Image data consisted of 74 patients including various CT examinations: brain, renal, liver, and lung scans.[10] The first acquisition was performed with standard dose and the second scan was acquired immediately after the first with

reduced radiation dose. All images were reconstructed by three different reconstruction algorithms: FBP, adaptive statistical iterative reconstruction (ASiR), and model-based iterative reconstruction (MBIR).

Using the noise addition tool, reduced-dose images were simulated from the standard dose images based on their dose reduction factor. The dose reduction factor was determined for each case by analyzing the dose values from the DICOM headers. The simulations were performed using a β value of 0.5 for both FBP and ASiR, and a β value of 0.17 for MBIR.

To assess the noise magnitude in the simulated and actual reduced-dose images of the patient scans, multiple regions of interest (ROIs) placed over different anatomical regions as shown in Fig. 3. A total of 442 ROIs were analyzed from three different anatomical regions based on the scan type. For brain scans, the ROIs were placed in the white matter, gray matter, and ventricle. For renal scans, the ROIs were placed in descending aorta, right kidney, and left kidney. For liver scans, the ROIs were placed in descending aorta, left kidney, and liver. For lung scans, the ROIs were placed in ascending aorta, descending aorta, and trachea. One renal case had one kidney, so only two ROIs were analyzed. Finally, the relative error of the noise magnitude (δ) was calculated using the following equation:

$$\delta\ (\%) = \frac{\sigma_{actual} - \sigma_{simulated}}{\sigma_{actual}} \times 100 \qquad (10)$$

Where σactual is the mean standard deviation of the actual reduced-dose images, and σsimulated is the mean standard deviation of the simulated reduced-dose images.

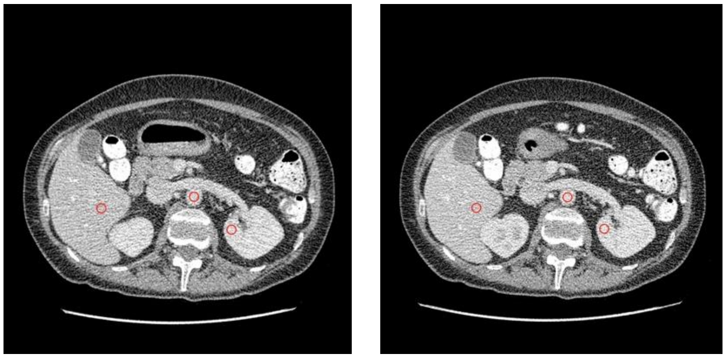

Fig. 3. A liver scan reconstructed with FBP, with ROIs at three anatomical regions: descending aorta, left kidney, and liver. (a) Actual reduced-dose image, and (b) simulated reduced-dose image.

2. *Smith TB, Abadi E, Sauer T, Fu W, Solomon J, Samei E. Development and validation of an automated methodology to assess perceptual in vivo noise texture in liver CT. J Med imaging (Bellingham, Wash). 2021;8(5):052113. doi:10.1117/1.JMI.8.5.052113*
3. *Christianson O, Winslow J, Frush DP, Samei E. Automated technique to measure noise in clinical CT examinations. Am J Roentgenol. 2015;205(1):W93-W99. doi:10.2214/AJR.14.13613*
4. *van Aarle W, Palenstijn WJ, Cant J, et al. Fast and flexible X-ray tomography using the ASTRA toolbox. Opt Express. 2016;24(22):25129. doi:10.1364/oe.24.025129*
5. *van Aarle W, Palenstijn WJ, De Beenhouwer J, et al. The ASTRA Toolbox: A platform for advanced algorithm development in electron tomography. Ultramicroscopy. 2015;157(2015):35-47. doi:10.1016/j.ultramic.2015.05.002*
6. *Wilson JM, Christianson OI, Richard S, Samei E. A methodology for image quality evaluation of advanced CT systems. Med Phys. 2013;40(3):7-11. doi:10.1118/1.4791645*
7. *Boedeker KL, Cooper VN, McNitt-Gray MF. Application of the noise power spectrum in modern diagnostic MDCT: Part I. Measurement of noise power spectra and noise equivalent quanta. Phys Med Biol. 2007;52(14):4027-4046. doi:10.1088/0031-9155/52/14/002*

8. *Dolly S, Chen HC, Anastasio M, Mutic S, Li H. Practical considerations for noise power spectra estimation for clinical CT scanners. J Appl Clin Med Phys. 2016;17(3):392-407. doi:10.1120/jacmp.v17i3.5841*
9. *Solomon J, Samei E. Quantum noise properties of CT images with anatomical textured backgrounds across reconstruction algorithms: FBP and SAFIRE. Med Phys. 2014;41(9). doi:10.1118/1.4893497*
10. *Solomon J, Mileto A, Nelson RC, Choudhury KR, Samei E. Quantitative features of liver lesions, lung nodules, and renal stones at multi-detector row CT examinations: Dependency on radiation dose and reconstruction algorithm. Radiology. 2016;279(1):185-194. doi:10.1148/radiol.2015150892.*

Table S1: Comparison of contrast and non-contrast scans

	Correctly classified malignancies (n, %)		
	Contrast scans (n=126)	Non-contrast scans (n=43)	*p-value*
Rule-in approach	73 (57.9%)	24 (55.8%)	*0.809*
Rule-out approach	120 (95.2%)	42 (97.7%)	*0.490*

Table S2: Comparison of solid and subsolid nodules

	Correctly classified malignancies (n, %)		
	Solid nodules (n=142)	Subsolid nodules (n=27)	*p-value*
Rule-in approach	85 (59.8%)	12 (44.4%)	*0.139*
Rule-out approach	137 (96.5%)	25 (92.6%)	*0.354*

Table S3: Dose parameters by imaging vendor

	Tube voltage (kV)	DLP (mGycm)	CTDI (Gy)
Siemens (n=102)	100 (100 –120)	247 (181 – 394)	10 (3 – 6)
Philips (n=27)	120 (100 – 120)	398 (265 – 671)	10 (6.1 – 22)
GE (n=25)	100 (100 – 120)	563 (286 – 1127)	15 (7 – 24)
Toshiba (n=15)	120 (120 – 120)	335 (177 – 429)	7 (4 – 10)

All values are depicted as median (interquartile range).

Table S4: Analysis by original effective dose

	Correctly classified malignancies (%)			
	Original dose	25%-dose	5%-dose	*p-value*
Dose group 1 (< 5 mSv, n=97)				
Rule-in approach	61.9%	58.8%	56.7%	*0.18*
Rule-out approach	97.9%	96.9%	94.8%	*0.1*
Dose group 2 (5-10 mSv, n=43)				
Rule-in approach	53.5%	55.8%	51.2%	*0.72*
Rule-out approach	93.0%	93.0%	90.7%	*0.37*
Dose group 3 (>10 mSv, n=24)				
Rule-in approach	50.0%	50.0%	37.5%	*0.05*
Rule-out approach	95.8%	100%	95.8%	*0.37*
